# Visual Hallucinations and Cognitive Impairment as the Initial Presentation of Severe Hypothyroidism in an Elderly Patient: A Case Report

**DOI:** 10.3390/geriatrics11030068

**Published:** 2026-06-05

**Authors:** Hongyu Li, Chenxiang Cao, Xiaoyi Wang, Jing Zhang, Wenhui Zhao

**Affiliations:** 1 Department of Endocrinology, Beijing Tsinghua Changgung Hospital, School of Clinical Medicine, Tsinghua Medicine, Tsinghua University, Beijing 102218, China; lhys02401@btch.edu.cn (H.L.); ccxa00369@btch.edu.cn (C.C.); wxya05531@btch.edu.cn (X.W.); 2 Department of Neurology, Beijing Tsinghua Changgung Hospital, School of Clinical Medicine, Tsinghua Medicine, Tsinghua University, Beijing 102218, China

**Keywords:** hypothyroidism, visual hallucinations, cognitive impairment, metabolic encephalopathy, dementia mimic

## Abstract

**Background:** Hypothyroidism is a prevalent endocrine disorder capable of affecting multiple organ systems. While somatic symptoms are common, prominent neuropsychiatric manifestations such as visual hallucinations and cognitive deficits are exceptionally rare as an initial presentation. This atypical clinical picture frequently leads to misdiagnosis as primary psychiatric disorders or neurodegenerative conditions. **Case Presentation:** We report the case of a 75-year-old male who presented with a three-month history of complex visual hallucinations and progressive cognitive decline. The hallucinations were notably dependent on environmental light. Initial assessments raised concerns about the neurodegenerative process. However, laboratory investigations revealed severe autoimmune hypothyroidism with high titers of anti-thyroid antibodies. Following timely levothyroxine replacement therapy, the patient achieved complete remission of visual hallucinations and significant cognitive recovery. **Conclusions:** This case illustrates that severe hypothyroidism can manifest as a reversible metabolic encephalopathy mimicking dementia. It underscores the importance of screening for thyroid dysfunction in elderly patients presenting with unexplained acute neuropsychiatric symptoms to ensure appropriate management.

## 1. Introduction

Hypothyroidism is a common endocrine disorder that involves multiple organ systems, yet it often lacks specific features in its early stages [1,2]. Although clinical manifestations typically include metabolic slowing, the occurrence of visual hallucinations and cognitive impairment as primary symptoms is rare [1,3]. Consequently, clinicians may misdiagnose this condition as a psychiatric disorder or a neurodegenerative disease [4,5].

Historically, the neuropsychiatric sequelae of hypothyroidism have been recognized since Asher’s classical description of ‘myxedema madness’ in 1949 [6]. While established literature indicates that approximately 5–15% of hypothyroid patients develop psychotic features, previous studies have predominantly focused on affective disorders, paranoid psychosis, or depressive stupor [5]. In contrast, complex visual hallucinations manifesting as the primary or sole presenting symptom remain exceptionally rare and are under-reported. Furthermore, when these hallucinations are accompanied by rapid cognitive decline, the clinical picture frequently mimics neurodegenerative conditions, leading to significant diagnostic delays. Review of previous cases suggests that such dementia mimics are often overlooked in the initial workup of elderly patients [7,8].

This report describes a case of autoimmune hypothyroidism in an elderly patient who presented primarily with complex, light-dependent visual hallucinations and cognitive dysfunction, clinically mimicking a neurodegenerative disorder. The distinctive features of this case include the predominance of visual hallucinations as an initial symptom, their clear dependence on environmental light, and substantial recovery after levothyroxine replacement therapy. We aim to enhance the recognition of this reversible metabolic presentation of severe hypothyroidism.

## 2. Case Presentation

A 75-year-old Asian male presented to our department with a chief complaint of visual hallucinations accompanied by memory decline and slowed reactions persisting for over three months. The symptoms began without a clear precipitating factor and exhibited marked environmental light dependency. The hallucinations primarily occurred in dark environments. After turning off the lights, the patient reported seeing unfamiliar small figures walking on the wall. He described these images as vivid and continuous, similar to watching television. These figures disappeared when the lights were turned on. Occasionally, he perceived furniture such as heating pipes and wardrobes as distorted or moving vertically.

During this period, his family noted fluctuating cognitive dysfunction characterized by significant short-term memory loss, bradyphrenia, and dulled responsiveness. The patient frequently forgot recent events, misplaced items, and struggled to maintain attention during complex daily activities. Associated symptoms included gait instability, difficulty initiating movement, and non-specific generalized fatigue. These symptoms occurred almost daily. He denied auditory hallucinations, headaches, dizziness, long-term memory impairment, or other behavioral symptoms. Since the onset of symptoms, the patient maintained a stable mental status but reported poor appetite with reduced food intake and low meat consumption. He also experienced poor sleep quality and constipation. There was no significant weight loss over the past month.

The patient had a high school education and was retired. His medical history included type 2 diabetes mellitus and hypertension, neither of which was managed regularly. He had no history of similar episodes, heart disease, tuberculosis, psychiatric disorders, head trauma, or psychotropic medication use. He denied smoking or alcohol consumption.

Upon admission to the Department of Neurology, physical examination revealed a body temperature of 36.3 °C, a pulse rate of 81 beats per minute, a respiratory rate of 20 breaths per minute, and a blood pressure of 125/87 mmHg. The patient was conscious but exhibited a sallow complexion and dulled responsiveness. While speech was clear and orientation to person, place, and time remained intact, bedside assessment indicated a gross deficit in short-term memory. Dermatologic examination showed dry, cool skin with poor elasticity. Generalized non-pitting edema was observed, predominantly affecting the face, hands, pretibial regions, and feet. Ocular examination revealed restricted vertical gaze in both eyes and left-sided ptosis. Pulmonary auscultation detected scattered moist rales, while cardiac examination demonstrated an enlarged cardiac border and distant heart sounds.

Neurologically, the patient displayed bradykinesia and a wide-based gait. While pain and temperature sensations were preserved, deep sensation was diminished in both lower extremities. Generalized hyporeflexia was noted, specifically characterized by a delayed relaxation phase of the Achilles tendon reflex. Coordination tests showed stability in the finger-to-nose test but instability in the heel-knee-shin test. The Romberg sign was positive. Muscle strength and tone were normal in all four limbs. Pathological reflexes were not elicited, and meningeal irritation signs were negative. The initial neurological impression included neurodegenerative changes, with dementia considered as a potential differential diagnosis. At this stage, a neurodegenerative disorder, particularly DLB, was considered because of the combination of recurrent visual hallucinations, fluctuating cognition, and gait disturbance. However, the subacute three-month course and the presence of systemic signs, including generalized edema, dry cool skin, delayed tendon reflex relaxation, and pericardial effusion, prompted further evaluation for reversible metabolic and endocrine causes.

The key diagnostic turning point was the thyroid function evaluation, which revealed profound primary hypothyroidism. The patient exhibited a markedly elevated thyroid-stimulating hormone level of 95.4 μIU/mL (reference range: 0.27–4.20 μIU/mL), alongside undetectable free thyroxine (0.5 pmol/L; reference range: 12.0–22.0 pmol/L) and free triiodothyronine (<0.6 pmol/L; reference range: 3.1–6.8 pmol/L). Additionally, thyroglobulin antibody titers were significantly elevated at >4000 KIU/L (reference range: <115 KIU/L), suggesting an autoimmune etiology, whereas thyroid peroxidase antibody remained within normal limits. Serum homocysteine levels were also elevated at 22.2 μmol/L (reference range: 5–15 μmol/L). Initial laboratory analysis showed that routine blood counts, liver function tests, coagulation profiles, tumor markers, neuron-specific enolase, routine stool and urine tests, infectious disease screening, folate, and vitamin B12 levels were all within normal ranges. Detailed results of the key abnormal laboratory findings on initial presentation are summarized in Table 1.

**Figure 1:**
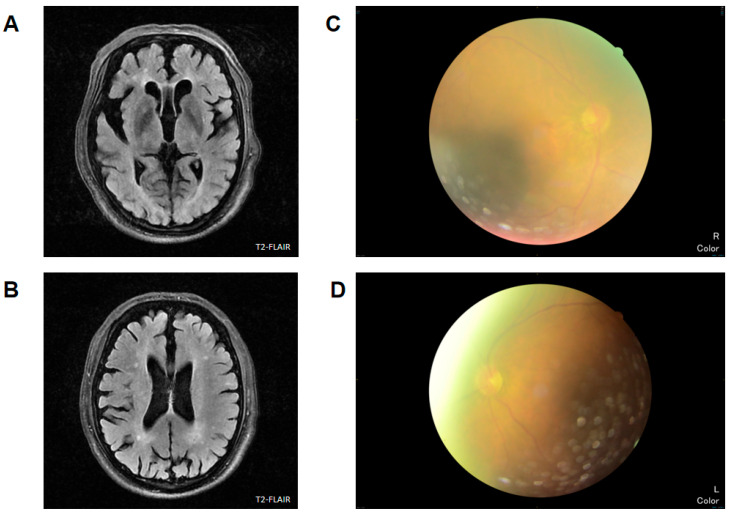
Cranial Magnetic Resonance Imaging (MRI) and Fundus Photography Findings. (**A**,**B**) Axial T2-FLAIR MRI sequences demonstrating scattered hyperintensities in the periventricular white matter (leukoaraiosis), consistent with age-related changes, without evidence of acute infarction or mass effect. (**C**,**D**) Bilateral fundus photographs showing clear optic disk margins without papilledema (excluding intracranial hypertension). No severe retinopathy was observed, helping to exclude Charles Bonnet Syndrome.

Cranial Magnetic Resonance Imaging revealed scattered demyelination in the periventricular white matter consistent with age-related changes. The occipital lobe structure remained intact, and there was no evidence of hippocampal atrophy, acute infarction, mass effect, or specific atrophy as shown in Figure 1A,B. Intracranial vascular imaging showed no severe stenosis in the vertebrobasilar system. Polysomnography indicated disordered sleep architecture accompanied by periodic limb movements but no specific neurodegenerative patterns or epileptiform discharges.

Cardiovascular assessment via echocardiography demonstrated a reduced left ventricular ejection fraction of 40% and massive pericardial effusion. Electrocardiography showed sinus rhythm with frequent premature ventricular complexes and ST-T wave changes. Chest Computed Tomography revealed bilateral pulmonary exudative changes, cardiomegaly, pericardial effusion, small bilateral pleural effusions, and potential pulmonary edema. Abdominal Computed Tomography indicated diffuse mild exudation and a small amount of fluid in the abdominopelvic cavity. Ophthalmologic examination showed no fundus hemorrhage or exudation, and the macular structure was intact, which excluded severe retinopathy and macular degeneration as seen in Figure 1C,D. Together, these findings made acute cerebrovascular disease, structural occipital pathology, epileptiform hallucinations, and major ophthalmologic causes of visual hallucinations less likely, thereby strengthening the suspicion of a systemic metabolic encephalopathy.

Cognitive assessment yielded a Mini-Mental State Examination (MMSE) score of 24 out of 30 and a Montreal Cognitive Assessment (MOCA) score of 16 out of 30. These results suggested moderate to severe cognitive impairment, with predominant deficits in visuospatial and executive functions. Detailed scoring is provided in Table 2.

The patient was diagnosed with severe hypothyroidism secondary to autoimmune thyroiditis. Given his advanced age and concurrent pericardial effusion, thyroid hormone replacement was initiated with levothyroxine sodium at a starting dose of 25 μg daily, which was gradually titrated to 75 μg daily. To address cardiac tamponade symptoms, pericardiocentesis was performed, resulting in rapid symptomatic relief.

**Figure 2:**
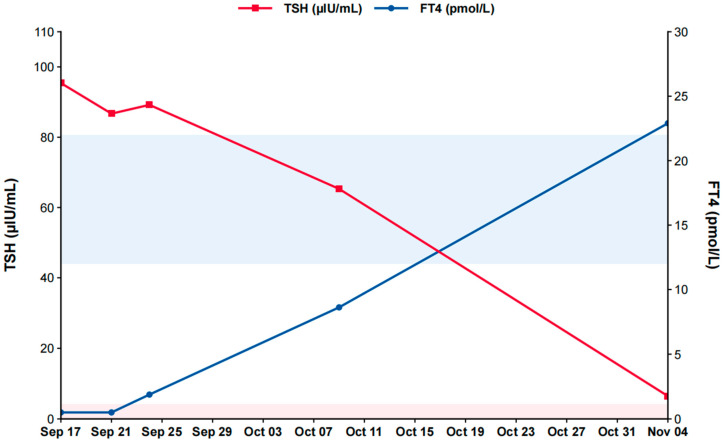
Temporal Trends in Thyroid Function Following Treatment. Temporal trends in thyroid function recovery. Serum Thyroid-Stimulating Hormone (TSH) levels are plotted on the left y-axis, and Free T4 (FT4) levels are plotted on the right y-axis. The x-axis represents the true time course, highlighting the rapid initial changes. The pink and blue shaded areas indicate the reference ranges for TSH (0.27–4.20 μIU/mL; left y-axis) and FT4 (12.0–22.0 pmol/L; right y-axis), respectively.

**Figure 3:**
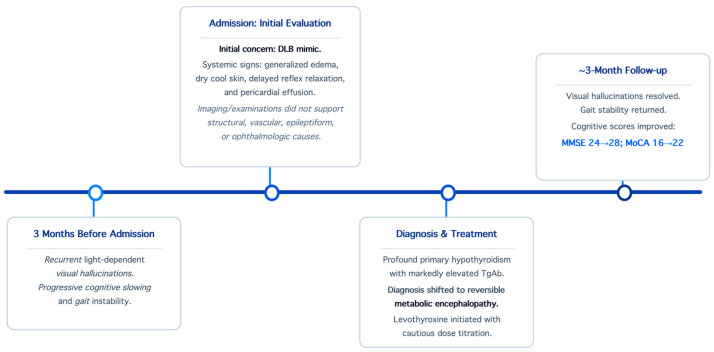
Clinical timeline of symptom onset, diagnostic evaluation, treatment, and recovery.

The patient attended follow-up visits at the endocrinology clinic at three and seven weeks post-discharge. Thyroid function tests showed progressive improvement as illustrated in Figure 2. Clinically, the visual hallucinations resolved completely, gait stability returned, and lower limb edema subsided. Approximately three months later, the Thyroid-Stimulating Hormone level decreased to 6.46 μIU/mL, and Free T4 normalized to 22.9 pmol/L. Creatine kinase levels also returned to the normal range. Repeat cognitive testing showed significant improvement, with a MMSE score of 28 out of 30 and a MOCA score of 22 out of 30. Detailed follow-up scores are listed in Table 2. This marked cognitive recovery supported the diagnosis of a reversible metabolic encephalopathy mimicking dementia. The clinical timeline of symptom onset, diagnostic evaluation, treatment initiation, and recovery is summarized in Figure 3.

During the three months before admission, the patient developed recurrent light-dependent visual hallucinations, progressive cognitive slowing, and gait instability. Initial evaluation raised concern for a dementia with Lewy bodies mimic. Systemic signs and thyroid function testing shifted the diagnostic focus toward severe hypothyroidism-induced reversible metabolic encephalopathy. After cautious levothyroxine dose titration, visual hallucinations resolved, gait stability returned, and cognitive scores improved at approximately three months.

## 3. Discussion

This case describes an elderly patient presenting with visual hallucinations and cognitive impairment as the initial manifestations of severe hypothyroidism. The diagnosis of autoimmune hypothyroidism-induced metabolic encephalopathy was established based on the presence of generalized myxedema, severe thyroid hormone deficiency, and the complete resolution of neuropsychiatric symptoms following thyroid hormone replacement.

To clarify the etiology, we integrated the prominent neuropsychiatric symptoms with systemic physical signs. The patient was an elderly male with a subacute onset of symptoms characterized by complex visual hallucinations, fluctuating cognitive decline, and an ataxic gait. Preliminary investigations did not support acute cerebral infarction, intracranial mass lesions, epileptic hallucinations, or central nervous system infections. Consequently, the differential diagnosis focused on distinguishing this condition from neurodegenerative diseases, vascular cognitive impairment, and Hashimoto’s encephalopathy [9,10,11,12,13].

Given the advanced age of the patient and the core complaints of visual hallucinations and cognitive deficits, we first considered neurodegenerative disorders such as Dementia with Lewy Bodies (DLB) and Alzheimer’s disease. Clinical criteria for DLB include fluctuating cognition, recurrent visual hallucinations, and parkinsonism [14,15]. Similarly, Alzheimer’s disease typically presents with episodic memory loss due to hippocampal damage and rarely manifests with vivid visual hallucinations in the early stages [16,17,18]. Both conditions generally exhibit an insidious onset with a chronic, irreversible course spanning years. In contrast, our patient presented with a rapid three-month progression and lacked definitive extrapyramidal signs. Neuropsychological testing indicated a frontal-subcortical pattern of impairment involving visuospatial and executive functions rather than the pure amnestic deficits typical of Alzheimer’s disease. Given the patient’s gait instability, positive Romberg sign, and heel-knee-shin test instability, these motor and balance findings were also clinically relevant to the cognitive workup. Balance assessment has been reported to correlate with functional decline and cognitive performance in older adults, suggesting its potential value as a complementary tool in the evaluation of cognitive deterioration [19]. Furthermore, the patient presented with significant pericardial effusion, a systemic manifestation that cannot be explained by a primary neurodegenerative disorder alone. Most importantly, neurodegenerative dementias are characterized by progressive deterioration [20,21]. The dramatic reversal of symptoms in this patient following metabolic correction provides the strongest evidence against a primary neurodegenerative etiology.

We also evaluated the possibility of vascular cognitive impairment given the history of diabetes and hypertension. Although Magnetic Resonance Imaging showed white matter demyelination suggesting chronic microvascular burden, vascular dementia typically follows a stepwise decline temporally correlated with stroke events [12,13,22]. This patient exhibited a subacute and continuous progression without a history of stroke or focal neurological deficits corresponding to specific vascular territories. Therefore, vascular factors alone could not account for the comprehensive clinical syndrome.

A critical diagnostic challenge involved distinguishing this case from Hashimoto’s encephalopathy, particularly given the high titers of anti-thyroid antibodies and cognitive dysfunction. Hashimoto’s encephalopathy is a diagnosis of exclusion often characterized by steroid responsiveness, whereas thyroid hormone replacement alone usually yields limited improvement [10,11,23,24]. In this case, the patient achieved rapid remission of neuropsychiatric symptoms with levothyroxine monotherapy without the use of corticosteroids. Pathologically, Hashimoto’s encephalopathy typically involves autoimmune-mediated cerebral microvasculitis or immune complex deposition, resulting in neuronal damage that is often independent of thyroid hormone levels [25,26,27]. The therapeutic response of this patient suggests that the symptoms were driven by metabolic hypothyroidism rather than the autoimmune inflammatory mechanisms typical of Hashimoto’s encephalopathy. Nonetheless, the possibility of Hashimoto’s encephalopathy cannot be completely excluded. Should neuropsychiatric symptoms recur, particularly under euthyroid conditions, Hashimoto’s encephalopathy and other autoimmune encephalitis syndromes should be reconsidered. In such a scenario, further evaluation including neuronal surface antibody testing, particularly anti-NMDAR and anti-LGI1 antibodies, would be warranted, and a corticosteroid trial could be considered after exclusion of infection and other contraindications [24,25]. Additionally, we excluded primary adrenal insufficiency, or Addison’s disease, as cortisol and adrenocorticotropic hormone levels were normal [28,29,30]. This exclusion was vital to ensure the safety of thyroid hormone replacement, as initiating treatment in the presence of uncorrected adrenal insufficiency could precipitate an adrenal crisis [31].

The exact pathophysiology underlying the neuropsychiatric manifestations of severe hypothyroidism remains incompletely understood. Contemporary research suggests that these manifestations may result from a multifaceted interplay of neurometabolic dysfunction, altered neurotransmitter regulation, and impaired sensory processing. Specifically, hypothyroidism has been associated with an imbalance of tyrosine hydroxylase in the anterior locus coeruleus and altered thyroid hormone receptor activity within the amygdala and hippocampus [32,33,34]. These brain regions are involved in arousal, emotional regulation, memory consolidation, and behavioral integration, which may help explain the bradyphrenia, fluctuating attention, and cognitive-behavioral deficits observed in our patient. Regarding the specific pathogenesis of visual hallucinations, it likely involves a synergistic interaction between metabolic encephalopathy and sensory deafferentation [35,36]. Severe hypothyroidism induces global cerebral hypometabolism, with Positron Emission Tomography studies showing reduced glucose utilization within the occipital visual cortex and association pathways [35,37,38]. In dark environments, this metabolic suppression, together with reduced external visual input, may increase deafferentation-related hypersensitivity and pathological release phenomena in the visual association cortex, a mechanism well established in Charles Bonnet syndrome and plausibly contributing to the patient’s light-dependent hallucinations [39,40,41,42,43]. This light-dependent pattern supports a release-hallucination mechanism, in which reduced afferent visual input unmasks internally generated visual percepts in a metabolically vulnerable visual network. Furthermore, the specific cognitive impairment profile observed here may have been exacerbated by hyperhomocysteinemia. Severe hypothyroidism can reduce methylenetetrahydrofolate reductase activity, leading to elevated homocysteine levels [44,45]. Hyperhomocysteinemia is known to induce oxidative stress and microcirculatory dysfunction, which may damage frontal-subcortical circuits [46,47,48,49]. The “double hit” of severe hypothyroidism and hyperhomocysteinemia may therefore have contributed to the executive and visuospatial deficits observed. This pattern of executive and visuospatial dysfunction may explain why the presentation clinically resembled a neurodegenerative syndrome rather than a purely psychiatric disorder. Although longitudinal assessment showed significant cognitive recovery, the MOCA score did not return to a fully normal range. This suggests that the metabolic encephalopathy may have been superimposed on a baseline of mild cognitive impairment. Following treatment, the patient remained free of hallucinations and maintained independent daily living skills. Taken together, these mechanisms provide a plausible explanation for the coexistence of light-dependent visual hallucinations, fluctuating cognition, and reversible metabolic encephalopathy in this patient.

This unique case contributes to the sparse literature documenting visual hallucinations and cognitive impairment as the initial presentation of severe hypothyroidism in a patient without a prior psychiatric history. The broad concept of “myxedema madness,” introduced as early as 1949, has traditionally emphasized affective or schizophrenia-like psychotic symptoms [6]. Although visual hallucinations may occur as accompanying symptoms in hypothyroidism-related neuropsychiatric syndromes, they have rarely been reported as the predominant initial complaint. Previous reports have more often described visual hallucinations in combination with broader psychotic symptoms, altered consciousness, or generalized cognitive slowing. In contrast, our patient sought medical attention primarily because of recurrent, complex visual hallucinations with a reproducible light-dependent pattern, accompanied by fluctuating cognitive decline and gait ataxia. The hallucinations occurred predominantly in dark environments and disappeared when the lights were turned on, making this presentation particularly unusual. Together, these findings closely mimicked a primary neurodegenerative process, most notably DLB [14]. The substantial clinical improvement after levothyroxine replacement further supports severe hypothyroidism as a reversible metabolic cause rather than a primary neurodegenerative disorder.

## 4. Conclusions

In conclusion, this case highlights a rare presentation of severe hypothyroidism manifesting primarily as visual hallucinations and cognitive impairment. The findings underscore that clinicians should include thyroid function testing in the screening protocol for elderly patients presenting with unexplained acute neuropsychiatric symptoms, even when clinical features mimic neurodegenerative diseases. Early recognition and appropriate levothyroxine replacement are essential for reversing neuropsychiatric symptoms and cognitive dysfunction, thereby supporting a favorable prognosis.

## Figures and Tables

**Table 1:** Key Abnormal Laboratory Findings on Initial Presentation.

Variable	Reference Range, Adults, This Hospital	On Initial Presentation, This Hospital
Thyroid Function		
Thyroid Stimulating Hormone (μIU/mL)	0.27–4.20	95.4
Free Thyroxine (pmol/L)	12.0–22.0	<0.5
Free Triiodothyronine (pmol/L)	3.1–6.8	<0.6
Thyroid Peroxidase Antibody (KIU/L)	<34	10.8
Thyroglobulin Antibody (KIU/L)	<115	>4000
Biochemistry and Electrolytes		
Sodium (mmol/L)	137–147	132.3
Potassium (mmol/L)	3.5–5.3	3.56
Chloride (mmol/L)	99–110	95.7
Carbon Dioxide (mmol/L)	22–29	23.6
Urea Nitrogen (mmol/L)	2.9–8.2	7.2
Creatinine (μmol/L)	67–109	126
Estimated Glomerular Filtration Rate (mL/min/1.73 m^2^)	>90	48.09
Glucose (mmol/L)	3.9–6.1	8.87
Lactate Dehydrogenase (U/L)	120–250	344
Homocysteine (μmol/L)	5–15	22.2
Cardiac Enzymes		
Creatine Kinase (U/L)	50–310	1171.4
Creatine Kinase-MB Isoenzyme (ng/mL)	0–6.36	10.53
Myoglobin (ng/mL)	0–80	112.0

**Table 2:** Cognitive assessment scores before and after levothyroxine replacement therapy.

Patient Profile				
Age: 75 years	Gender: Male	Marital Status: Married	Occupation: Retired	Education Level: High School
**Domain**		**Baseline**		**Follow-up (3 months)**
Mini-Mental State Examination				
Orientation to Time		4		5
Orientation to Place		5		4
Immediate Recall		1		3
Attention and Calculation		5		5
Delayed Recall		1		2
Naming		2		2
Repetition		1		1
Reading		1		3
Auditory Comprehension		2		1
Writing		1		1
Copying		1		1
Total Score		24		28
Montreal Cognitive Assessment				
Visuospatial/Executive Function (5 points)		1		3
Naming (3 points)		3		3
Memory/Delayed Recall (5 points)		0		1
Attention (6 points)		5		6
Language (3 points)		1		1
Abstraction (2 points)		0		1
Orientation (6 points)		5		6
Education Adjustment (1 point)		1		1
Total Score		16		22

## Data Availability

The data presented in this study are available upon request from the corresponding author. The data are not publicly available due to patient privacy and ethical restrictions.

## Abbreviations

The following abbreviations are used in this manuscript:

DLB	Dementia with Lewy Bodies
FT4	Free Thyroxine
FT3	Free Triiodothyronine
TSH	Thyroid-Stimulating Hormone
MRI	Magnetic Resonance Imaging
MMSE	Mini-Mental State Examination
MOCA	Montreal Cognitive Assessment
PET	Positron Emission Tomography

## References

[1] Taylor P.N., Medici M.M., Hubalewska-Dydejczyk A., Boelaert K. (2024). Hypothyroidism. Lancet.

[2] Chaker L., Papaleontiou M. (2025). Hypothyroidism: A Review. JAMA.

[3] Lekurwale V., Acharya S., Shukla S., Kumar S. (2023). Neuropsychiatric Manifestations of Thyroid Diseases. Cureus.

[4] Reed K., Bland R.C. (1977). Masked “myxedema madness”. Acta Psychiatr. Scand..

[5] Heinrich T.W., Grahm G. (2003). Hypothyroidism Presenting as Psychosis: Myxedema Madness Revisited. Prim. Care Companion J. Clin. Psychiatry.

[6] Asher R. (1949). Myxoedematous madness. Br. Med. J..

[7] Alšauskė S.V., Liseckienė I., Verkauskienė R. (2024). The Association of Thyroid Disease with Risk of Dementia and Cognitive Impairment: A Systematic Review. Medicina.

[8] Davis J.D., Tremont G. (2007). Neuropsychiatric aspects of hypothyroidism and treatment reversibility. Minerva Endocrinol..

[9] Livingston G., Huntley J., Sommerlad A., Ames D., Ballard C., Banerjee S., Brayne C., Burns A., Cohen-Mansfield J., Cooper C. (2020). Dementia prevention, intervention, and care: 2020 report of the Lancet Commission. Lancet.

[10] Chaudhuri J., Mukherjee A., Chakravarty A. (2023). Hashimoto’s Encephalopathy: Case Series and Literature Review. Curr. Neurol. Neurosci. Rep..

[11] Montagna G., Imperiali M., Agazzi P., D’Aurizio F., Tozzoli R., Feldt-Rasmussen U., Giovanella L. (2016). Hashimoto’s encephalopathy: A rare proteiform disorder. Autoimmun. Rev..

[12] Hainsworth A.H., Markus H.S., Schneider J.A. (2024). Cerebral Small Vessel Disease, Hypertension, and Vascular Contributions to Cognitive Impairment and Dementia. Hypertension.

[13] Rundek T., Tolea M., Ariko T., Fagerli E.A., Camargo C.J. (2022). Vascular Cognitive Impairment (VCI). Neurotherapeutics.

[14] McKeith I.G., Boeve B.F., Dickson D.W., Halliday G., Taylor J.-P., Weintraub D., Aarsland D., Galvin J., Attems J., Ballard C.G. (2017). Diagnosis and management of dementia with Lewy bodies: Fourth consensus report of the DLB Consortium. Neurology.

[15] Devenyi R.A., Hamedani A.G. (2024). Visual dysfunction in dementia with Lewy bodies. Curr. Neurol. Neurosci. Rep..

[16] Knopman D.S., Amieva H., Petersen R.C., Chételat G., Holtzman D.M., Hyman B.T., Nixon R.A., Jones D.T. (2021). Alzheimer disease. Nat. Rev. Dis. Primers.

[17] Jack C.R., Andrews J.S., Beach T.G., Buracchio T., Dunn B., Graf A., Hansson O., Ho C., Jagust W., McDade E. (2024). Revised criteria for diagnosis and staging of Alzheimer’s disease: Alzheimer’s Association Workgroup. Alzheimer’s Dement..

[18] Weintraub D., Aarsland D., Chaudhuri K.R., Dobkin R.D., Leentjens A.F., Rodriguez-Violante M., Schrag A. (2022). The neuropsychiatry of Parkinson’s disease: Advances and challenges. Lancet Neurol..

[19] Biasin F., Ceolin C., Celli S., Terziotti C., Raffaelli C., Bontempi C., Devita M., De Rui M., Sergi G., Coin A. (2023). Interrelation between functional decline and dementia: The potential role of balance assessment. Hum. Mov. Sci..

[20] Sorbi S., Hort J., Erkinjuntti T., Fladby T., Gainotti G., Gurvit H., Nacmias B., Pasquier F., Popescu B.O., Rektorova I. (2012). EFNS-ENS Guidelines on the diagnosis and management of disorders associated with dementia. Eur. J. Neurol..

[21] Revi M. (2020). Alzheimer’s Disease Therapeutic Approaches. Adv. Exp. Med. Biol..

[22] The VasCog-2-WSO Criteria Consortium (2025). Revised Diagnostic Criteria for Vascular Cognitive Impairment and Dementia. JAMA Neurol..

[23] Chaigne B., Beaufils E., Jouan Y., Magnant J., Maillot F., Constans T., Hommet C., Mondon K. (2012). Hashimoto’s encephalopathy. Rev. Méd. Interne.

[24] Endres D., Leypoldt F., Bechter K., Hasan A., Steiner J., Domschke K., Wandinger K.-P., Falkai P., Arolt V., Stich O. (2020). Autoimmune encephalitis as a differential diagnosis of schizophreniform psychosis. Eur. Arch. Psychiatry Clin. Neurosci..

[25] Mocellin R., Walterfang M., Velakoulis D. (2007). Hashimoto’s encephalopathy: Epidemiology, pathogenesis and management. CNS Drugs.

[26] Varley J.A., Strippel C., Handel A., Irani S.R. (2023). Autoimmune encephalitis: Recent clinical and biological advances. J. Neurol..

[27] Sy A.J.R., Anlacan V.M.M., Yu A.B., Jamora R.D.G. (2025). New onset steroid-responsive Hashimoto’s encephalopathy in the older population: A scoping review. Neurol. Sci..

[28] Bancos I., Hahner S., Tomlinson J., Arlt W. (2015). Diagnosis and management of adrenal insufficiency. Lancet Diabetes Endocrinol..

[29] Bornstein S.R., Allolio B., Arlt W., Barthel A., Don-Wauchope A., Hammer G.D., Husebye E.S., Merke D.P., Murad M.H., Stratakis C.A. (2016). Diagnosis and Treatment of Primary Adrenal Insufficiency: An Endocrine Society Guideline. J. Clin. Endocrinol. Metab..

[30] Betterle C., Presotto F., Furmaniak J. (2019). Epidemiology, pathogenesis, and diagnosis of Addison’s disease in adults. J. Endocrinol. Investig..

[31] Jonklaas J., Bianco A.C., Bauer A.J., Burman K.D., Cappola A.R., Celi F.S., Cooper D.S., Kim B.W., Peeters R.P., Rosenthal M.S. (2014). Guidelines for the treatment of hypothyroidism: Prepared by the ATA task force. Thyroid.

[32] Crocker A.D., Overstreet D.H., Crocker J.M. (1986). Hypothyroidism leads to increased dopamine receptor sensitivity and concentration. Pharmacol. Biochem. Behav..

[33] Claustre J., Balende C., Pujol J.F. (1996). Influence of the thyroid hormone status on tyrosine hydroxylase. Neurochem. Int..

[34] Omri M., Ferhi M., Lentz N., Oliveira Galvao M., Hamm O. (2024). Myxedema Psychosis: Diagnostic Challenges and Management Strategies. Cureus.

[35] Pak K., Kim M., Kim K., Kim B.H., Kim S.-J., Kim I.J. (2020). Cerebral glucose metabolism and Cerebral blood flow in thyroid dysfunction. Sci. Rep..

[36] Sawicka-Gutaj N., Zawalna N., Gut P., Ruchała M. (2022). Relationship between thyroid hormones and CNS metabolism. Pharmacol. Rep..

[37] Wang H., Tan Z., Zheng Q., Yu J. (2018). Metabolic Brain Network Analysis of Hypothyroidism Symptom Based on PET of Rats. Mol. Imaging Biol..

[38] Constant E.L., de Volder A.G., Ivanoiu A., Bol A., Labar D., Seghers A., Cosnard G., Melin J., Daumerie C. (2001). Cerebral blood flow and glucose metabolism in hypothyroidism: A PET study. J. Clin. Endocrinol. Metab..

[39] Manford M., Andermann F. (1998). Complex visual hallucinations. Clinical and neurobiological insights. Brain.

[40] O’Brien J., Taylor J.P., Ballard C., A Barker R., Bradley C., Burns A., Collerton D., Dave S., Dudley R., Francis P. (2020). Visual hallucinations in neurological and ophthalmological disease: Pathophysiology and management. J. Neurol. Neurosurg. Psychiatry.

[41] Marschall T.M., Brederoo S.G., Ćurčić-Blake B., Sommer I.E.C. (2020). Deafferentation as a cause of hallucinations. Curr. Opin. Psychiatry.

[42] Collerton D., Barnes J., Diederich N.J., Dudley R., Ffytche D., Friston K., Goetz C.G., Goldman J.G., Jardri R., Kulisevsky J. (2023). Understanding visual hallucinations: A new synthesis. Neurosci. Biobehav. Rev..

[43] Merabet L.B., Maguire D., Warde A., Alterescu K., Stickgold R., Pascual-Leone A. (2004). Visual hallucinations during prolonged blindfolding in sighted subjects. J. Neuro-Ophthalmol..

[44] Orzechowska-Pawiłojć A., Lewczuk A., Sworczak K. (2005). The influence of thyroid hormones on homocysteine and atherosclerotic vascular disease. Endokrynol. Pol..

[45] Cui L., Wang F., Li C., Liu F., Wang H., Zhao J. (2025). Homocysteine and thyroid diseases. Front. Endocrinol..

[46] Dayal S., Arning E., Bottiglieri T., BogEr R.H., Sigmund C.D., Faraci F.M., Lentz S.R. (2004). Cerebral vascular dysfunction mediated by superoxide in hyperhomocysteinemic mice. Stroke.

[47] Lominadze D., Tyagi N., Sen U., Ovechkin A., Tyagi S.C. (2012). Homocysteine alters cerebral microvascular integrity by antagonizing GABA-A receptor. Mol. Cell. Biochem..

[48] Dufouil C., Alpérovitch A., Ducros V., Tzourio C. (2003). Homocysteine, white matter hyperintensities, and cognition in healthy elderly people. Ann. Neurol..

[49] Song H., Bharadwaj P.K., Raichlen D.A., Habeck C.G., Grilli M.D., Huentelman M.J., Hishaw G.A., Trouard T.P., Alexander G.E. (2024). Cortical lobar volume reductions associated with homocysteine-related brain atrophy. Front. Aging Neurosci..

